# Impact of question order on prioritisation of outcomes in the development of a core outcome set: a randomised controlled trial

**DOI:** 10.1186/s13063-017-2405-6

**Published:** 2018-01-25

**Authors:** Sara T. Brookes, Katy A. Chalmers, Kerry N. L. Avery, Karen Coulman, Jane M. Blazeby, on behalf of the ROMIO study group, C Paul Barham, Richard Berrisford, Jenny Donovan, Jackie Elliott, Stephen Falk, Robert Goldin, George Hanna, Andrew Hollowood, Sian Noble, Grant Sanders, Tim Wheatley

**Affiliations:** 1 The MRC ConDuCT-II Hub for Trials Methodology Research, Population Health Sciences, Bristol Medical School, University of Bristol, Bristol, UK; 2 Cancer Research UK Clinical Trials Unit (CRCTU), Institute of Cancer and Genomic Sciences, University of Birmingham, Edgbaston, Birmingham, B15 2TT UK; 3 Division of Surgery, Head and Neck, University Hospitals Bristol NHS Foundation Trust, Bristol, UK

**Keywords:** Core outcome set, Delphi, Question order, Context effects

## Abstract

**Background:**

Core outcome set (COS) developers increasingly employ Delphi surveys to elicit stakeholders’ opinions of which outcomes to measure and report in trials of a particular condition or intervention. Research outside of Delphi surveys and COS development demonstrates that question order can affect response rates and lead to ‘context effects’, where prior questions determine an item’s meaning and influence responses. This study examined the impact of question order within a Delphi survey for a COS for oesophageal cancer surgery.

**Methods:**

A randomised controlled trial was nested within the Delphi survey. Patients and health professionals were randomised to receive a survey including clinical and patient-reported outcomes (PROs), where the PRO section appeared first or last. Participants rated (1–9) the importance of 68 items for inclusion in a COS (ratings 7–9 considered ‘essential’). Analyses considered the impact of question order on: (1) survey response rates; (2) participants’ responses; and (3) items retained at end of the survey.

**Results:**

In total, 116 patients and 71 professionals returned completed surveys. Question order did not affect response rates among patients, but fewer professionals responded when clinical items appeared first (difference = 31.3%, 95% confidence interval [CI] = 13.6–48.9%, *P* = 0.001). Question order led to different context effects within patients and professionals. While patients rated clinical items highly, irrespective of question order, more PROs were rated essential when appearing last rather than first (difference = 23.7%, 95% CI = 10.5–40.8%). Among professionals, the greatest impact was on clinical items; a higher percentage rated essential when appearing last (difference = 11.6%, 95% CI = 0.0–23.3%). An interaction between question order and the percentage of PRO/clinical items rated essential was observed for patients (*P* = 0.025) but not professionals (*P* = 0.357). Items retained for further consideration at the end of the survey were dependent on question order, with discordant items (retained by one question order group only) observed in patients (18/68 [26%]) and professionals (20/68 [29%]).

**Conclusions:**

In the development of a COS, participants’ ratings of potential outcomes within a Delphi survey depend on the context (order) in which the outcomes are asked, consequently impacting on the final COS. Initial piloting is recommended with consideration of the randomisation of items in the survey to reduce potential bias.

**Trial registration:**

The randomised controlled trial reported within this paper was nested within the development of a core outcome set to investigate processes in core outcome set development. Outcomes were not health-related and trial registration was not therefore applicable.

**Electronic supplementary material:**

The online version of this article (doi:10.1186/s13063-017-2405-6) contains supplementary material, which is available to authorized users.

## Background

Core outcome sets (COS) are recommended for use in clinical effectiveness trials to reduce heterogeneity of reported outcomes and aid data synthesis across similar trials, enhancing evidence-based medicine and reducing research waste [1–4]. A COS is an agreed minimum set of outcomes to be measured and reported in all trials of a particular condition or intervention [4]. Their development requires consensus methodology to establish outcomes considered most essential to patients and health professionals. One increasingly used approach is a Delphi survey [5–7], where participants are required to anonymously rate the importance of a long list of potential outcomes in sequential (postal or electronic) surveys or ‘rounds’ [8]. Feedback from each round is presented in the subsequent round such that participants can consider the opinions of others before re-rating items. The results of the Delphi inform any further consensus methods (such as a consensus meeting [9–11]) and the final COS. Guidelines exist for the Delphi process, within the context of a COS [4, 5, 12] and more widely elsewhere [13–17], with emphases on selection of stakeholders, number of rounds, presentation of feedback and criteria for consensus. Far less focus has been awarded to the actual design of the Delphi survey itself, which has been criticised as often being poorly formulated [17, 18].

One issue that may be important within Delphi surveys is the ordering of questions and the potential for question order to impact on both overall survey response rate and individual responses to questions. Within Social and Health Sciences, there are numerous publications relating to the design of questionnaires or surveys and question order is frequently discussed [19–21]. The choice of initial items may influence a respondent’s willingness or motivation to complete a survey since early items may shape a respondent’s understanding of what the survey is about [19]. Previous literature, including randomised studies, has demonstrated mixed effects in terms of overall survey response rate [22–24]. In terms of actual responses to questions, when items are not asked in isolation it is likely (at least for some individuals) that responses to earlier questions will be used as a comparative standard by which to respond; consequently, the order of questions (or the ‘context’ in which questions are asked) may influence responses [21, 25]. This phenomenon is often referred to as a ‘context effect’ [19, 20, 25]. Indeed, such effects have been observed in numerous randomised and non-randomised studies [19–21, 25–29]. While focus has commonly been on the ordering of general and specific questions (with the recommendation that the general question should precede the specific, since the specific are more likely to influence the general than vice versa) [20, 26, 28–31], effects have also been observed with the ordering of two or more similarly specific items [21, 25]. In order to explore question order effects, Moore [25] suggests a comparison of responses to two questions in the non-comparative context (when question asked first) and the comparative context (when question asked after another one). When responses to the two questions become more similar in the comparative than the non-comparative context we observe what is termed a consistency effect [21, 25], where respondents attempt to be consistent with their earlier responses. When responses become more different in the comparative context we observe a contrast effect [21, 25], respondents emphasising differences between items rather than the similarities.

In the context of Delphi surveys, we are only aware of one publication warning of such context effects [16]. Delphi surveys, constructed for COS development, generally include attitudinal questions, asking respondents to rate the importance of a succession of specific outcomes that may be valued differently. In such a setting it seems plausible that question order and context effects may lead to a significant bias [16], which is likely to influence the resulting COS.

This study explored the impact of question order within a Delphi survey used in the development of a COS for oesophageal cancer surgery. The following hypotheses were considered:

1. The ordering of items impacts on Delphi survey response rates;
2. The ordering of items effects participants’ responses (context effects); and the effect differs among patients and health professionals;
3. The ordering of items influences the items retained at the end of the first Delphi round.

## Methods

This methodological work employed a parallel randomised controlled trial, nested within a Delphi survey. The Delphi survey aimed to prioritise a ‘long list’ of outcomes to inform a COS (finalised at a subsequent consensus meeting) for clinical effectiveness trials of oesophageal cancer surgery. The development of the COS has been described in detail elsewhere [32]. An exhaustive ‘long list’ of outcomes was identified from a literature review, clinical audit and patient interviews [33–36]. Overlapping outcomes were merged and categorised into health domains and included as individual items in the survey. Items consisted of 38 patient-reported outcomes (PROs) and 30 clinical outcomes. Patients and health professionals were asked to rate the importance of each item for inclusion in a COS, from 1 (not essential) to 9 (absolutely essential). Two versions of the survey were created. In version 1, PROs were presented first and the clinical outcomes last (termed ‘PRO first’), and in version 2, the clinical outcomes were presented first and the PROs last (‘PRO last’). For both versions, the items within the PRO and clinical sections were presented in identical order. PROs were grouped under a single heading of ‘quality of life after discharge from hospital’; clinical outcomes were grouped into headed sections of ‘benefits of oesophageal cancer surgery’, ‘in-hospital events’ and ‘events after hospital discharge’. In both versions items were written in lay terms with medical terms in brackets.

While the Delphi process consisted of two survey rounds (round 2 including feedback for each item retained from round 1), this study focused on the impact of question order on round 1 responses. This enabled the impact of question order to be explored in isolation, rather than being compounded by any effect of dropping items or presenting feedback from the previous round.

### Stakeholder groups

Patients who had undergone oesophagectomy were identified from one of two UK hospital trusts (University Hospitals Bristol NHS Foundation Trust and Plymouth Hospitals NHS Trust). After establishing patients’ status, living patients were posted an invitation letter and information leaflet and asked to return a consent form indicating willingness to participate in the study. Consenting patients were then sent a postal survey with a pre-paid return envelope. Health professionals from relevant disciplines and clinical backgrounds (oesophagogastric surgeons and clinical nurse specialists) were identified from the membership of the Association of Upper Gastro Intestinal Surgeons of Great Britain and Ireland [32]. These professionals were notified by email about the study and sent a survey through the post with a pre-paid return envelope. Reminders were sent via post or email (for patients and professionals, respectively) to non-responders.

### Randomisation

Participants were randomised, using a computer-generated schedule, to receive version 1 (PRO first) or version 2 (PRO last) in a 1:1 ratio. The schedule was generated separately for patients and professionals due to the different approaches to recruitment. For patients, who were recruited dynamically, block randomisation was stratified by centre. Once a consent form was received and logged on the database, the patient received the next allocation. All identified health professionals were randomised before they were notified of the study. In this instance, professionals were assigned an identification number and then simple randomisation was used to determine which received PRO first surveys and which received PRO last. This allocation schedule was used (within a mail-merge) to automatically generate the allocated survey for each participant.

### Statistical analyses

#### Sample size

This nested study was opportunistic in nature, with the sample size determined by the numbers of patients and professionals participating in the Delphi process. Statistical hypothesis testing is, therefore, largely exploratory.

The following analyses were employed to address the study hypotheses. All statistical analyses were performed in Stata version 14 [37].

##### 1. Impact on Delphi survey response rates

Response rates were calculated for each version of the survey (PRO first or PRO last) and for each stakeholder group (patients and health professionals), with the total number of surveys sent out to each sub-group (version and stakeholder group) as the denominator. The proportion responding was compared between randomisation groups, separately for patients and health professionals, with a Chi-square test or Fisher’s exact test as appropriate. The difference in proportions, 95% confidence interval (CI) and *P* value are reported. Demographic data were not available for non-responders hence we were unable to explore potential causal factors for non-response other than randomisation group.

##### 2. Effect on participants’ responses (context effects) among patients and health professionals

The percentage of PROs rated essential (scored 7–9) [4] and the percentage of clinical items rated essential (7–9) was calculated for each participant. Distributional checks were carried out. Considering patients and professionals separately, two-by-two tables were generated presenting the mean (or median if data skewed) percentage of PRO and clinical items rated essential in the non-comparative context, when each was presented first in the survey, and in the comparative context, when each was presented last (and could therefore be rated in comparison to those items presented first). The difference between PROs and clinical items was calculated for both the non-comparative and comparative context (with 95% CI). The difference between appearing first and last in the survey was also calculated for both PROs and clinical items (with 95% CI). Visual examination of these tables provided insights into potential context effects [25]. While individual statistical tests could be carried out to ascertain if each of these four differences are ‘significantly’ different from zero, a more appropriate approach (and one which reduces the number of statistical tests) is to formally test for an interaction between the percentage of PROs/clinical items rated essential and question order. An appropriate analytical approach is afforded by the equivalence of the design of this study to that of a simple AB vs BA crossover trial [38].

In the analysis of a simple crossover trial, investigators often consider the potential of a treatment-period interaction, that is, where the effectiveness of treatment A compared to B is dependent on the order that treatments are received? Within the current study, a ‘treatment-period’ or PRO/clinical-position interaction is present if respondents rate PRO/clinical items differently if they come first or if they follow the other item type. To explore this, the average percentage of PROs and clinical items rated essential were calculated for each participant and the difference in means (or medians if data are skewed) compared between the randomisation groups [38]. In the absence of an interaction participants’ average percentage of items rated essential would be the same regardless of question order. The resulting distribution was examined by stakeholder and randomisation group. Unpaired t-tests or Mann–Whitney tests were then employed as appropriate (and 95% CI calculated), comparing randomisation groups. Analyses were carried out separately for patients and health professionals.

Analyses were repeated considering the median ratings given to PROs and clinical outcomes (rather than the percentage rated essential) and the consistency of results examined. In additional post-hoc analyses surgeons and clinical nurse specialists were considered separately.

##### 3. Influence on the items retained at the end of the Delphi round 1

In the development of the COS, at the end of round 1 items were retained for round 2 if they were rated 7–9 by 70% or more of respondents and 1–3 by < 15% [32]. These criteria were considered separately for the two stakeholder groups and items retained if they met the criteria for patients and/or professionals. For the purposes of this paper, these criteria were additionally applied within each randomisation group separately. Two-by-two contingency tables categorised the number of items retained by: (1) both the PRO first and PRO last group; (2) the PRO first group only; (3) the PRO last group only; and (4) neither group. The percentage of discordant items, retained by one randomisation group but not the other, was calculated.

## Results

**Fig. 1:**
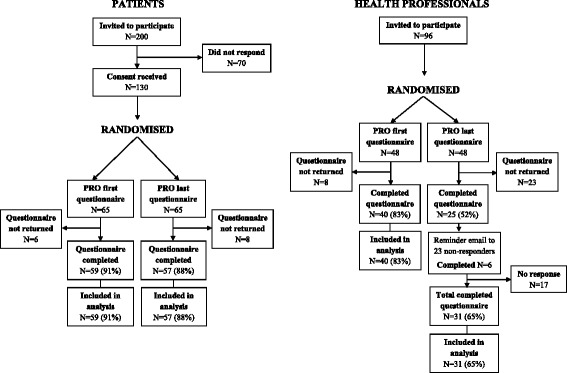
Flow diagram

The round 1 survey contained 68 items (38 PROs and 30 clinical items). In total, 200 patients were invited to participate in the Delphi survey, of whom 130 (65%) provided consent and were allocated to and sent either a PRO first or PRO last survey. Ninety-six health professionals were identified and randomised and the allocated survey sent for completion (Fig. 1).

### 1. Impact on Delphi survey response rates

A higher overall rate of questionnaire return, ignoring randomisation group, was observed within patients (89.2%) as compared to health professionals (74.0%). This is likely a consequence of the questionnaire only being sent to patients who had already provided consent. Response rates among patients were high; 59 (91%) and 57 (88%) within the PRO first and PRO last groups, respectively, demonstrating no difference between the question order randomisation groups (difference = 3.1%, 95% CI = −7.6–13.7%, *P* = 0.572). Among health professionals, however, a difference was observed between question order groups. Within the PRO first group, 40 (83%) surveys were completed compared to only 23 (52%) among the PRO last group (difference = 31.3%, 95% CI = 13.6–48.9%, *P* = 0.001). Due to study constraints, reminders were only sent to the professionals allocated to PRO last group given the very poor response rate. Even after a reminder, the response rate in this group remained significantly lower (31 [65%]) than in the PRO first group (difference = 18.8%, 95% CI = 1.6–35.9%, *P* = 0.036).

### Baseline comparison of randomisation groups

Table 1 presents baseline characteristics of those responding to the round 1 survey. The question order groups are largely similar except for a lower percentage of male patients and a higher percentage of younger professionals responding to the PRO last survey.

**Table 1:** Baseline demographics of participants completing questionnaire

Stakeholder group	PRO first	PRO last
Patients	n = 59	n = 57
Male, n (%)	52 (88.1)	42 (73.7)
Age, mean (SD)^a^	66.7 (7.2)	66.3 (8.9)
Education, n (%)^b^		
None, GCSE	28 (50.0)	30 (54.5)
A level, further education	17 (30.3)	17 (30.9)
Other^c^	11 (19.6)	8 (14.5)
Employment, n (%)		
Working full-time	8 (13.6)	10 (17.5)
Retired	40 (67.8)	36 (63.2)
Other	11 (18.6)	11 (19.3)
Years since surgery, median (IQR)^d^	1.3 (0.7–2.3)	1.4 (0.6–2.3)
Hospital stay < 2 weeks, n (%)^e^	35 (60.3)	37 (67.3)
Health professionals	n = 40	n = 31
Male, n (%)	29 (72.5)	23 (74.2)
Age, n (%) (years)		
≤40	3 (7.5)	7 (22.6)
41–50	18 (45.0)	14 (45.2)
51–60	18 (45.0)	6 (19.4)
>60	1 (2.5)	4 (12.9)
Job title, n (%)		
Consultant surgeon	30 (75.0)	22 (71.0)
Surgical registrar	1 (2.5)	1 (3.2)
Clinical specialist nurse	9 (22.5)	8 (25.8)

^a^Age missing for one ‘PRO first’ patient

^b^Education missing for three ‘PRO first’ patients and two ‘PRO last’ patients

^c^Majority of ‘other’ are vocational qualifications with insufficient detail for classification

^d^Years since surgery missing for three ‘PRO first’ patients and three ‘PRO last’ patients

^e^Hospital stay missing for one ‘PRO first’ patient and two ‘PRO last’ patients

#### 2. Effect on participants’ responses (context effects) among patients and health professionals

The percentage of PROs rated essential (scored 7–9) and the percentage of clinical items rated essential was calculated for each participant. In order to explore potential context effects (such as consistency and contrast), PRO and clinical ratings were considered in both a non-comparative and comparative context and among patients and professionals separately. Distributions were heavily negatively skewed; since appropriate transformations would significantly hinder interpretation, median and IQRs are reported.

#### Patients

**Fig. 2:**
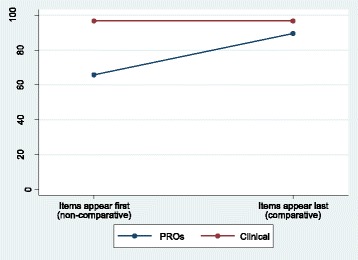
Patients: percentage of items rated essential within the non-comparative and comparative context (a consistency effect)

Table 2 summarises (as median and IQR) the percentage of PROs and clinical items rated essential by patients when presented first (non-comparative) and last (comparative) [25]. Patients rated clinical items very highly, irrespective of question order (96.7% rated essential in both the PRO first and PRO last groups). However, far more PROs were rated essential when they appeared after clinical items than when they appeared first (difference = 23.7%, 95% CI = 10.5–40.8%). When asked about PROs first, patients on average rated 66% of PROs as essential; when the other half of the patients were asked about clinical outcomes first, they rated on average 97% of clinical outcomes essential. Hence, in a non-comparative context, participants rate on average 31% more clinical items than PROs as essential. However, in the comparative context, the difference (in favour of clinical items) is reduced to just 7%. This demonstrates a consistency effect – the difference between PROs and clinical outcomes becomes smaller in the comparative context. This effect can perhaps more clearly be seen when considered graphically in Fig. 2.

**Table 2:** Patients: percentage of items rated essential within the non-comparative and comparative context (a consistency effect)

Context of rating	Percentage of items rated essential by a participant, median (IQR)		Difference in medians (clinical minus PROs) (95% CI)^a^
	PROs (38 items)	Clinical (30 items)	
Appearing first (non-comparative)	65.8 (47.4–89.5)	96.7 (73.3–100.0)	30.9 (11.8–39.2)
Appearing last (comparative)	89.5 (60.5–97.4)	96.7 (63.3–100.0)	7.2 (−1.4–13.2)
Difference in medians (last minus first) (95% CI)^a^	23.7 (10.5–40.8)	0.0 (0.0–20.0)	−23.7

Number of patients: PRO first n = 59; PRO last n = 57

^a^Bias-corrected bootstrap 95% CI

The average percentage of PRO and clinical items rated essential by each participant was calculated (to test for a PRO/clinical-position interaction). The resulting distribution was heavily negatively skewed and parametric tests comparing the randomisation groups demonstrated that assumptions for such a test were violated. Non-parametric tests were therefore performed and evidence of a PRO/clinical-position interaction effect observed (Mann–Whitney *P* = 0.0250) (Table 4).

#### Health professionals

**Fig. 3:**
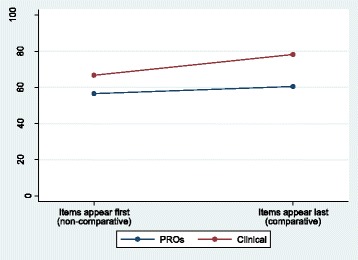
Health professionals: percentage of items rated essential within the non-comparative and comparative context (a contrast effect)

Health professionals rated a higher percentage of both PROs and clinical items as essential when they appeared last in the survey (Table 3), with the greatest impact seen for clinical items (11.6% more items rated essential when they appeared last, 95% CI = 0.0–23.3%). In the non-comparative context (PRO/clinical items presented first), professionals on average rated 57% PROs as essential compared to 67% clinical outcomes – a 10% difference. In the comparative context (PRO/clinical items presented last) the percentage of essential PROs increased marginally to 61% and clinical outcomes increased to 78%, resulting in a greater difference between the two types of items of 17%. In this instance, we have a contrast effect because the difference increases in the comparative context. Again, this contrast effect can be seen more clearly in Fig. 3. However, there was less evidence of a PRO/clinical-position interaction in this instance (Mann–Whitney *P* = 0.3567, Table 4), the observed effects likely to be due to chance.

**Table 3:** Health professionals: percentage of items rated essential within the non-comparative and comparative context (a contrast effect)

Context of rating	Percentage of items rated essential by a participant, median (IQR)		Difference in medians (clinical minus PROs) (95% CI)^a^
	PROs (38 items)	Clinical (30 items)	
Appearing first (non-comparative)	56.6 (42.1–85.5)	66.7 (60.0–83.3)	10.1 (−7.7–22.1)
Appearing last (comparative)	60.5 (26.3–89.5)	78.3 (66.7–86.7)	17.8 (−7.5–43.2)
Difference in medians (last minus first) (95% CI)^a^	3.9 (−23.7–31.6)	11.6 (0.0–23.3)	+7.7

Number of professionals: PRO first n = 40; PRO last n = 31

^a^Bias-corrected bootstrap 95% CI

**Table 4:** Item type-position interaction effects

	Average^a^ percentage of PROs and clinical items rated essential, median (IQR)		Difference in medians (95% CI)^b^	*P* value^c^
	PRO first	PRO last		
Patients	80.9 (59.7 to 93.4)	92.1 (61.4 to 98.7)	11.2 (−2.5 to 19.7)	0.025
Health professionals	68.2 (58.3 to 81.6)	70.0 (42.3 to 82.0)	1.8 (−14.8 to 13.3)	0.357

^a^Average calculated for each participant as ([% essential PROs] + [% essential clinical])/2

^b^Bias-corrected bootstrap 95% CIs

^c^*P* values derived from Mann–Whitney tests

Given the opposite context effects observed, analysis ignoring stakeholder group has not been presented here. In brief, such analysis produced consistent results to those seen for patients, a likely consequence of the larger sample size in this participant group. Within this study, 76% of health professionals were surgeons and only 24% specialist nurses (Table 1). Post-hoc analyses considered potential context effects separately for nurses and surgeons. Unlike surgeons and patients, nurses rated PROs as more essential than clinical items. In the non-comparative context, 20% more PROs on average were rated essential than clinical items, compared to only 5% more in the comparative context – demonstrating a consistency effect within nurses (Additional file 1: Table S1). The number of participants was, however, small within this group. Additional file 2: Table S2 presents the results for surgeons only; unsurprisingly, patterns were similar to those for all professionals combined.

Analyses considering the median ratings given to PROs and clinical outcomes (rather than the percentage rated essential) demonstrated the same effects as those reported above.

##### 3. Influence on the items retained at the end of the Delphi round 1

Applying pre-specified criteria for retaining items at the end of round 1, discordant items were observed where an item was retained by one question order group and not the other (Table 5). The degree of discrepancy was dependent on item type (PRO or clinical) and stakeholder group. The greatest discrepancy within patients was in terms of PROs (42% discordant items) and within professionals it was in terms of clinical items (37% discordant items). If items were retained when the pre-specified criteria were met by patients and/or professionals (criteria used for the overall development of the oesophageal surgery COS [32]), there remained 24% (16/68) discordant items between the question order groups (Table 5). Hence, question order impacts on the items retained for round 2.

**Table 5:** Number of items retained at end of round 1 by patients and health professionals

Stakeholder group	Participants (n)		Outcome type	Items retained at end of round 1^a^, n (%)				Discordant items (%)
	PRO first	PRO last		Retained by both groups	Retained by PRO first only	Retained by PRO last only	Retained by neither group	
Patients	59	57	PRO	14/38	0/38	16/38	8/38	42.1
			Clinical	28/30	2/30	0/30	0/30	13.3
Health professionals	40	31	PRO	5/38	7/38	2/38	24/38	23.7
			Clinical	12/30	10/30	1/30	7/30	36.7
All^b^	99	88	PRO	17/38	1/38	13/38	7/38	36.8
			Clinical	28/30	2/30	0/30	0/30	6.7

^a^Items were retained by each stakeholder group if they were scored 7–9 by 70% or more and 1–3 by < 15%

^b^For ‘all’ participants, items were retained if scored 7–9 by 70% or more and 1–3 by < 15% within either stakeholder group

In the development of the oesophageal surgery COS (within which this methodological work is nested), the investigators combined the randomisation groups at the end of round 1 to determine which items to retain for all participants [32]. In round 2 (in which participants received a survey with questions in the same order as their round 1 survey), question order effects were again considered and similar patterns observed as in round 1.

## Discussion

This methodological work examined the impact of question order within the first round of a Delphi survey to inform a COS for oesophageal cancer resection surgery. Question order did not impact on response rates within patients; however, fewer health professionals responded to the survey when clinical items appeared first and PRO items last. While participants consistently rated clinical items more essential than PROs (irrespective of question order or stakeholder group), context effects (where prior questions affect responses to later questions) were observed among both stakeholder groups, though the direction of these effects differed. Patients inflated the importance of PROs when rating them last in the survey, being more consistent with their earlier judgments regarding clinical items (consistency effect), whereas professionals inflated the importance of clinical items when they appeared last, emphasising their greater importance compared to PROs previously rated (contrast effect). Moreover, this study observed that question-order impacted on items retained at the end of round 1 (based on pre-specified criteria), which will ultimately influence the final COS and, therefore, is of utmost importance. Given these findings, we would strongly recommend that potential question order effects are considered when designing and implementing a Delphi survey for the development of a COS.

The results of this study agree with previous literature within survey research (including both non-randomised and randomised studies) and extend it to Delphi surveys and COS development. The majority of research into question order effects has dealt with behavioural or factual items that are verifiable. In Delphi surveys for COS development participants are asked attitudinal questions, being required to rate how important they feel different outcomes are relative to each other. In this situation, it is implicit that participants consider items in comparison to previous items; hence, context effects are perhaps more likely than in other settings [16, 39].

Items presented at the beginning of a survey may motivate or demotivate an individual to respond [19]. In this study, health professionals appear to have been less motivated to respond if clinical items appeared first. One may hypothesise that if PROs appear first, a professional might feel strongly compelled to express their opinion that these are not the most important items, whereas if clinical items (such as survival) appear first that same professional might feel less driven (or less need) to respond. Within this study, opposite context effects were seen within patients and professionals. This agrees with Birckart [40] who argues that consistency effects (what he terms ‘carryover’) are more likely when respondents feel they are moderately knowledgeable (such as patients), whereas contrast effects (‘backfire’) are more likely when respondents are highly knowledgeable (such as health professionals in the relevant field).

Recent research has demonstrated that different types of health professionals value different outcomes and that each group should be adequately represented [41, 42]. Within this study, 76% of health professionals were surgeons (consultant and registrar) and only 24% specialist nurses. Additional post-hoc analyses demonstrated that surgeons and nurses prioritised different outcomes. Moreover, question order resulted in different context effects within these two groups of health professionals. While the number of nurses in this analysis was small, given the observed differences we would support recent recommendations that different health professionals should be considered as separate panels during the Delphi process [42].

In the current study, some degree of imbalance was observed between the randomisation groups in terms of the gender of patients and the age of health professionals. This may be due to chance or it may (at least partially) be due to certain individuals being more or less likely to respond to the different versions of the survey (PRO first and PRO last). For example, women may be more likely (or men less likely) and younger professionals more likely (or older professionals less likely) to respond when clinical items are first (PRO last). Previous authors have suggested that the magnitude of order effects may depend on participant demographics [26]; however, few studies have provided empirical evidence. McFarland found no evidence of question order effect varying with sex or education [29], but a later study observed order effects among less-educated respondents only [30, 40]. We are not aware of any studies that have specifically considered age. Further exploration within this current study examined male and female patients and younger and older professionals separately (Additional files 3, 4, 5 and 6: Tables S3-S6). Patterns were largely consistent, with perhaps a greater consistency effect within women than men and a greater contrast effect within younger rather than older professionals; however, numbers of participants were small within individual groups.

Patients and health professionals were the only stakeholder groups included in this study and it is possible that different question order effects may occur in other groups such as methodologists or regulators. However, patients and health professionals are considered the most essential stakeholders to include in the development of a COS [5] and are likely to make up a large majority, if not all, of the Delphi participants. This study included participants only from the UK and within a single disease setting; it is important, therefore, to repeat this study in other countries and settings. In addition, not all Delphi surveys drop items (deemed less essential) at the end of each round, instead retaining all items until the end of the final round. However, in such a scenario it is highly likely that if context effects are present, due to the design of the survey, they will impact on responses in all rounds and the subsequent final COS.

This is the first study we are aware of investigating question order within a Delphi for COS development and, while exploratory in nature, it provides the best evidence at present, that such effects should be considered in this setting. Initial piloting of the Delphi survey may be valuable in identifying potential question order effects and we would recommend that this is always done. Cognitive interviews, such as ‘Think Aloud’ [43], carried out while individuals complete the survey with different orderings of items, may help identify if and how responses are influenced by earlier items. Previous survey research offers potential recommendations to reduce potential question-order effects. Question-order effects are assumed to arise because items similar in content influence one another [26]; this has led to the suggestion that such items could be separated with ‘buffer’ questions [27, 39, 44]. One potential within a Delphi survey for a COS, such as that described in this current paper, might be to alternate clinical and PRO items. However, this may interrupt the flow of the survey, making it less coherent [26], and guidelines suggest that items within the same theme should be grouped together [21]. Future research should explore this approach further.

An alternative approach for COS development is to randomise participants to receive surveys with different question orders and then combine the responses across the different surveys. Indeed, within the field of survey research this approach was recommended as long as 40 years ago [45] and has been reiterated since [16, 19, 20, 28]. The idea here is that when the data are combined across all randomised participants (as in the development of the oesophageal COS) question-order effects will be ‘cancelled out’ or at least diminished. This current paper has only considered the ordering of two ‘blocks’ of items (PRO and clinical), which produces only two different randomised versions. We have not considered potential order effects within those ‘blocks’ which may also exist. Again, initial piloting with cognitive interviews may help identify the extent of randomisation required. While it would be plausible to randomise items within ‘blocks’, it may be more logistically challenging, although this is likely to be easier for an electronic Delphi survey than a postal one. This should be explored further.

Within the context of crossover trials, when strong period-treatment interactions are observed, one recommendation is to use data from the first period only from each of the randomisation groups [38]. This has also been recommended within survey research, where question order has been randomised, in the belief that responses to questions asked in the non-comparative context are a better representation of an individual’s true feelings [29]. However, in the context of prioritising potential outcomes for a COS, it could be argued that an outcome cannot be rated without consideration of other outcomes and so the comparative context may be more appropriate.

While context effects were observed in this exploratory study, further work is needed to replicate and confirm our findings within the development of other core sets. It is, however, plausible that question order may, to some extent, have impacted on previously developed COSs which have employed Delphi surveys. A crucial part of the development of a COS is its subsequent periodic review in order to validate the COS and ensure outcomes are still important [4]. For COSs initially developed without consideration of question order, such a review would afford the opportunity to consider such potential effects. This research does not invalidate previously developed COSs but offers a potential enhancement to the review and updating of COSs and the development of future COSs.

In addition to initial piloting of the Delphi survey, in the absence of further research we would recommend that question order within a Delphi survey is randomised, at least in terms of the presentation of clinical and patient-reported outcomes, and that the responses are then combined across randomisation groups to inform the final COS.

Finally, while this study has considered the use of a Delphi survey to inform a COS, question order is also likely to have an impact in other forms of consensus methodology such as the Nominal Group Technique or less-structured consensus meetings. While these approaches do not generally incorporate a formal questionnaire, items for discussion are still presented to participants in some order. Without running multiple meetings, it is difficult to envisage how randomisation could be utilised in this scenario. The Delphi method enables randomisation of question order and impact of question order to be examined empirically afterwards.

## Conclusions

Core outcome set (COS) developers are increasingly employing Delphi surveys to elicit stakeholders’ opinions as to the most essential outcomes to measure and report in trials of a particular condition or intervention. There is currently little guidance as to the optimal structure of such surveys. This paper demonstrates that participants’ ratings of potential outcomes within a Delphi survey depend on the order in which the outcomes are presented. Initial piloting of such surveys is recommended with consideration of the randomisation of items in the Delphi survey.

## Additional files

Additional file 1: Table S1. Nurses: percentage of items rated essential within the non-comparative and comparative context (a consistency effect). (DOCX 13 kb)

Additional file 2: Table S2. Surgeons: percentage of items rated essential within the non-comparative and comparative context (a contrast effect). (DOCX 13 kb)

Additional file 3: Table S3. Male patients: percentage of items rated essential within the non-comparative and comparative context (a consistency effect). (DOCX 12 kb)

Additional file 4: Table S4. Female patients: percentage of items rated essential within the non-comparative and comparative context (a consistency effect). (DOCX 12 kb)

Additional file 5: Table S5. Health professionals (aged ≤ 50 years): percentage of items rated essential within the non-comparative and comparative context (a contrast effect). (DOCX 12 kb)

Additional file 6: Table S6. Health professionals (aged > 50 years): percentage of items rated essential within the non-comparative and comparative context (a contrast effect). (DOCX 12 kb)

## Abbreviations

CI: Confidence interval
COS: Core outcome set
GI: Gastrointestinal
IQR: Interquartile range
PRO: Patient-reported outcome
REC: Research ethics committee
