# Supplementary material for: Impact of question order on prioritisation of outcomes in the development of a core outcome set: a randomised controlled trial
Source: Trials. 2018 Jan 25;19:66. doi: 10.1186/s13063-017-2405-6 (PMC5784591; doi:10.1186/s13063-017-2405-6)
Supplement: Supplementary file 1 — Nurses: percentage of items rated essential within the non-comparative and comparative context (a consistency effect). (DOCX 13 kb) [file 13063_2017_2405_MOESM1_ESM.docx]

**Supplementary Table 1:** Nurses - percentage of items rated essential within the non-comparative and comparative context (a consistency effect)

| Context of rating | Percentage of items rated essential by a participant, median (IQR) | | Difference in medians (clinical minus PROs), (95% CI)^a^ |
| --- | --- | --- | --- |
|  | PROs (38 items) | Clinical (30 items) |  |
| Appearing first  (non-comparative) | 86.8 (71.1-94.7) | 66.7 (55.0-81.7) | -20.2 (-31.8, 16.1) |
| Appearing last (comparative) | 92.1 (82.9-97.4) | 86.6 (73.3-100.0) | -5.4 (-25.4, 17.1) |
| Difference in medians (last minus first), (95% CI) ^a^ | 5.2  (-7.9, 34.2) | 20.0  (-10.0, 41.7) | +14.8 |

Number of nurses: PRO first N=9; PRO last N=8

^a^Bias-corrected bootstrap 95% confidence interval
