# Supplementary material for: Impact of question order on prioritisation of outcomes in the development of a core outcome set: a randomised controlled trial
Source: Trials. 2018 Jan 25;19:66. doi: 10.1186/s13063-017-2405-6 (PMC5784591; doi:10.1186/s13063-017-2405-6)
Supplement: Supplementary file 2 — Surgeons: percentage of items rated essential within the non-comparative and comparative context (a contrast effect). (DOCX 13 kb) [file 13063_2017_2405_MOESM2_ESM.docx]

**Supplementary Table 2:** Surgeons - percentage of items rated essential within the non-comparative and comparative context (a contrast effect)

| Context of rating | Percentage of items rated essential by a participant, median (IQR) | | Difference in medians (clinical minus PROs), (95% CI)^a^ |
| --- | --- | --- | --- |
|  | PROs (38 items) | Clinical (30 items) |  |
| Appearing first  (non-comparative) | 52.6 (31.6-73.7) | 66.7 (60.0-83.3) | 14.0 (-0.6, 28.7) |
| Appearing last (comparative) | 44.7 (18.4-73.7) | 76.7 (62.1-86.7) | 31.9 (6.1, 46.8) |
| Difference in medians (last minus first), (95% CI) ^a^ | -7.9  (-27.6, 18.4) | 10.0  (-12.3, 16.7) | +17.9 |

Number of surgeons: PRO first N=31; PRO last N=23

^a^Bias-corrected bootstrap 95% confidence interval
