# Supplementary material for: Impact of question order on prioritisation of outcomes in the development of a core outcome set: a randomised controlled trial
Source: Trials. 2018 Jan 25;19:66. doi: 10.1186/s13063-017-2405-6 (PMC5784591; doi:10.1186/s13063-017-2405-6)
Supplement: Supplementary file 3 — Male patients: percentage of items rated essential within the non-comparative and comparative context (a consistency effect). (DOCX 12 kb) [file 13063_2017_2405_MOESM3_ESM.docx]

**Supplementary Table 3:** Male patients - percentage of items rated essential within the non-comparative and comparative context (a consistency effect)

| Context of rating | Percentage of items rated essential by a participant, median (IQR) | | Difference in medians (clinical minus PROs), (95% CI)^a^ |
| --- | --- | --- | --- |
|  | PROs (38 items) | Clinical (30 items) |  |
| Appearing first  (non-comparative) | 65.8 (47.4, 86.8) | 96.7 (73.3, 100.0) | 30.9  (14.4, 43.9) |
| Appearing last (comparative) | 86.8 (68.4, 97.4) | 96.7 (65.0, 100.0) | 9.9  (4.6, 24.3) |
| Difference in medians (last minus first), (95% CI) | 21.0  (9.8, 39.4) | 0.0  (-1.7, 20.0) | -21.0 |

Number of male patients: PRO first N=52; PRO last N=42

^a^ Bias-corrected bootstrap 95% confidence interval
