# Supplementary material for: Impact of question order on prioritisation of outcomes in the development of a core outcome set: a randomised controlled trial
Source: Trials. 2018 Jan 25;19:66. doi: 10.1186/s13063-017-2405-6 (PMC5784591; doi:10.1186/s13063-017-2405-6)
Supplement: Supplementary file 4 — Female patients: percentage of items rated essential within the non-comparative and comparative context (a consistency effect). (DOCX 12 kb) [file 13063_2017_2405_MOESM4_ESM.docx]

**Supplementary Table 4:** Female patients - percentage of items rated essential within the non-comparative and comparative context (a consistency effect)

| Context of rating | Percentage of items rated essential by a participant, median (IQR) | | Difference in medians (clinical minus PROs), (95% CI)^a^ |
| --- | --- | --- | --- |
|  | PROs (38 items) | Clinical (30 items) |  |
| Appearing first  (non-comparative) | 44.7 (15.8-94.7) | 100 (65.5-100) | 55.3 (1.9, 100.0) |
| Appearing last (comparative) | 97.3 (55.3-100) | 96.7 (37.5-100) | -0.6 (-62.5, 39.5) |
| Difference in medians (last minus first), (95% CI) | 52.6  (-5.2, 94.7) | -3.3  (-62.5, 23.3) | -55.9 |

Number of female patients: PRO first N=7; PRO last N=15

^a^ Bias-corrected bootstrap 95% confidence interval
