# Supplementary material for: Impact of question order on prioritisation of outcomes in the development of a core outcome set: a randomised controlled trial
Source: Trials. 2018 Jan 25;19:66. doi: 10.1186/s13063-017-2405-6 (PMC5784591; doi:10.1186/s13063-017-2405-6)
Supplement: Supplementary file 5 — Health professionals (aged ≤ 50 years): percentage of items rated essential within the non-comparative and comparative context (a contrast effect). (DOCX 12 kb) [file 13063_2017_2405_MOESM5_ESM.docx]

**Supplementary Table 5:** Health professionals (aged <=50) - percentage of items rated essential within the non-comparative and comparative context (a contrast effect)

| Context of rating | Percentage of items rated essential by a participant, median (IQR) | | Difference in medians (clinical minus PROs), (95% CI)^a^ |
| --- | --- | --- | --- |
|  | PROs (38 items) | Clinical (30 items) |  |
| Appearing first  (non-comparative) | 55.3 (39.5-84.2) | 66.7 (60.0-80.0) | 11.4 (-12.2, 27.7) |
| Appearing last (comparative) | 57.9 (31.6-84.2) | 76.7 (62.1-86.7) | 18.8 (-14.9, 43.9) |
| Difference in medians (last minus first), (95% CI) | 2.6  (-28.9, 31.6) | 10.0  (-10.0, 21.7) | +7.4 |

Number of younger professionals: PRO first N=21; PRO last N=21

^a^ Bias-corrected bootstrap 95% confidence interval
