# Supplementary material for: Impact of question order on prioritisation of outcomes in the development of a core outcome set: a randomised controlled trial
Source: Trials. 2018 Jan 25;19:66. doi: 10.1186/s13063-017-2405-6 (PMC5784591; doi:10.1186/s13063-017-2405-6)
Supplement: Supplementary file 6 — Health professionals (aged > 50 years): percentage of items rated essential within the non-comparative and comparative context (a contrast effect). (DOCX 12 kb) [file 13063_2017_2405_MOESM6_ESM.docx]

**Supplementary Table 6:** Health professionals (aged >50) - percentage of items rated essential within the non-comparative and comparative context (a contrast effect)

| Context of rating | Percentage of items rated essential by a participant, median (IQR) | | Difference in medians (clinical minus PROs), (95% CI)^a^ |
| --- | --- | --- | --- |
|  | PROs (38 items) | Clinical (30 items) |  |
| Appearing first  (non-comparative) | 60.5 (44.7-89.5) | 75.0 (60.0-96.7) | 14.5 (-16.1, 41.4) |
| Appearing last (comparative) | 76.7 (26.3-92.1) | 80.0 (73.3-86.7) | 3.3 (-16.1, 55.4) |
| Difference in medians (last minus first), (95% CI) ^a^ | 16.2  (-36.8, 44.7) | 5.0  (-15.0, 43.3) | -11.2 |

Number of older professionals: PRO first N=19; PRO last N=10

^a^Bias-corrected bootstrap 95% confidence interval
